# Phylogenetic and temporal dynamics of human immunodeficiency virus type 1 CRF01_AE and CRF07_BC among recently infected antiretroviral therapy-naïve men who have sex with men in Jiangsu province, China, 2012 to 2015: A molecular epidemiology-based study

**DOI:** 10.1097/MD.0000000000009826

**Published:** 2018-02-09

**Authors:** Yue Yang, Xiu-Ping Zhao, Hua-Chun Zou, Min-Jie Chu, Ping Zhong, Xiao-Shan Li, Xiao-Yan Li, Yu-Hui Yu, Ke-Xin Zhu, Yu-Jia Chen, Fei Xia, Bo-Wen Zhu, Luan-Qi Ruan, Yi-Ning Bao, Xun Zhuang

**Affiliations:** a Department of Epidemiology and Health Statistics, School of Public Health, Nantong University, Nantong; b Department of Infection Management Office, the First Affiliated Hospital with Nanjing Medical University, Jiangsu Province Hospital, Nanjing, Jiangsu Province; c Department of AIDS and STD, Shanghai Municipal Center for Disease Control and Prevention; Shanghai Municipal Institute for Preventive Medicine, Shanghai; d Department of AIDS and STD, Suzhou Center for Disease Prevention and Control, Suzhou, Jiangsu Province; e School of Public Health, Sun Yat-sen University, Guangzhou, Guangdong Province, China; f Kirby Institute, University of New South Wales, Sydney, Australia; g Teaching and Research Office of Epidemiology and Health Statistics, School of Public Health, Southeast University, Nanjing, Jiangsu Province; h Ningbo Medical Center Lihuili Eastern Hospital; Taipei Medical University Ningbo Medical Center, Ningbo, Zhejiang Province, China.

**Keywords:** China, CRF01_AE, CRF07_BC, HIV-1, men who have sex with men, most recent common ancestor (MRCA)

## Abstract

The prevalence and incidence of human immunodeficiency virus type 1 (HIV-1) among men who have sex with men (MSM) are on the rise throughout China. With a large population of MSM, Jiangsu Province is facing an escalating HIV-1 epidemic.

The aim of this study was to explore the phylogenetic and temporal dynamics of HIV-1 CRF01_AE and CRF07_BC among antiretroviral therapy (ART)-naïve MSM recently infected with HIV-1 in Jiangsu Province.

We recruited MSM in Jiangsu Province (Suzhou, Wuxi, Nantong, Taizhou and Yancheng) 2012 to 2015. We collected information on demographics and sexual behaviors and a blood sample for HIV genome RNA extraction, RT-PCR amplification, and DNA sequencing. Multiple alignments were made using Gene Cutter, with the selected reference sequences of various subtypes/recombinants from the Los Alamos HIV-1 database. Phylogenetic and Bayesian evolutionary analysis was performed by MEGA version 6.0, Fasttree v2.1.7. and BEAST v1.6.2. Categorical variables were analyzed using *χ*^2^ test (or Fisher exact test where necessary). *χ*^2^ test with trend was used to assess the evolution of HIV-1 subtype distribution over time. All data were analyzed using SPSS20.0 software package (IBM Company, New York, NY).

HIV-1 phylogenetic analysis revealed a broad viral diversity including CRF01_AE (60.06%), CRF07_BC (22.29%), subtype B (5.88%), CRF67_01B (5.26%), CRF68_01B (2.79%), CRF55_01B (1.55%), CRF59_01B (0.93%), and CRF08_BC (0.62%). Two unique recombination forms (URFs) (0.62%) were also detected. Four epidemic clusters and 1 major cluster in CRF01_AE and CRF07_BC were identified. The introduction of CRF01_AE strain (2001) was earlier than CRF07_BC strain (2004) into MSM resided in Jiangsu based on the time of the most recent common ancestor.

Our study demonstrated HIV-1 subtype diversity among ART-naïve MSM recently infected with HIV-1 in Jiangsu. We first depicted the spatiotemporal dynamics, traced the dates of origin for the HIV-1 CRF01_AE/07_BC strains and made inference for the effective population size among newly infected ART-naïve MSM in Jiangsu from 2012 to 2015. A real-time surveillance of HIV-1 viral diversity and phylodynamics of epidemic cluster would be of great value to the monitoring of the epidemic and control of transmission, improvement of antiretroviral therapy strategies, and design of vaccines.

## 1 Introduction

China is facing an enormous challenge for controlling the rapid spread of the HIV-1 epidemic through sexual transmission. According to the official report, since the first HIV case found in 1985, approximately 177,000 HIV-infected people had died and the number of people living with HIV/AIDS had reached 575,000 by the end of 2015. Nearly 100,000 people were newly diagnosed between January and October 2015.^[1]^ The proportion of MSM in newly diagnosed cases saw the sharp increase overtime: from 2.5% in 2006 to 27.2% in 2015. In 2013, a large-scale cross-sectional survey involving MSM from 61 cities in China revealed a pooled HIV prevalence of 4.9%.^[2]^ This rate had further increased to 8.0% in 2015.^[1]^ As a coastal province with booming economy and cultural diversity in eastern China, bordering Shanghai to the south, Jiangsu has been attracting an increasing population of migrants, including MSM. From January to September 2014, 54.5% of the newly diagnosed cases in Jiangsu were MSM,^[3]^ much higher than the national proportion of 25.8% in 2014.^[4]^

In Jiangsu, the main subtypes of HIV-1 among all risk populations was CRF01_AE (56.8%), followed by subtype B (19.9%).^[5]^ For MSM, the main subtypes were CRF01_AE (63.6%), CRF07_BC (18.2%), B (9.1%), and CRF08_BC (9.1%).^[6]^ Nationwide molecular epidemiologic surveys indicated that CRF01_AE strain, initially prevailing in the heterosexual risk individuals in southwestern border provinces and eastern coastal areas,^[7,8]^ had quickly overtaken subtype B among MSM during the past few years.^[9–11]^ Meanwhile, CRF07_BC strain, the other dominating subtype among injection drug users (IDUs),^[12–14]^ was also reported to be spreading among MSM in some provinces.^[11,15–17]^ Surprisingly, CRF55_01B strain, a newly identified recombinant virus, has been rapidly disseminated among MSM and heterosexuals in Shenzhen in southern China and elsewhere (Hong Kong, Dongguan, Hunan, Shanghai, Beijing, and so on) at the end of 2012.^[18,19]^ Therefore, an ever-increasing migrant population and a sharply rising trend of HIV-1 infection in MSM in Jiangsu will inevitably evolve the HIV-1 subtype diversity.

Our study aimed to explore the phylogenetic and temporal dynamics of HIV-1 CRF01_AE and CRF07_BC among recently infected antiretroviral therapy (ART)-naïve MSM in Jiangsu Province, using established phylogenetic and evolutionary analysis methods.

## 2 Materials and methods

### 2.1 Ethics

The study, including design, consent, and laboratory procedures, was reviewed and approved by the Institutional Review Board at the Human Medical Research Ethics Committee of the municipal Center for Disease Control and Prevention in each of the 5 study cities (SZCDC, WXCDC, NTCDC, TZCDC, YCCDC).

### 2.2 Study subjects

We retrospectively investigated 323 MSM from initial 1265 HIV-1-infected patient samples and their related demographic data in 5 major cities (Suzhou, Wuxi, Nantong, Taizhou, and Yancheng) of Jiangsu Province from 2012 to 2015, from which the sequences were isolated and analyzed based on our sentinel surveillance and drug resistance database.

To be eligible, participants must be diagnosed (Western blot confirmation) annually for HIV-1 infection, have resided in Jiangsu in the past 3 months, be highly active antiretroviral therapy-naïve, have a high-quality sequence of at least 1000 nucleotides based on the *pol* region.

Three criteria for determination of HIV-1 primary or recent infection have been extensively applied. They are defined by diagnosis during clinically defined acute HIV infection (http://www.shcs.ch/56-definitions2.1) or during recent infection defined by seroconversion (<1 year between last-negative and first-positive HIV test), or by an ambiguous nucleotide count of <0.5% (in the first year) in a baseline in bulk sequencing of HIV-1 *pol* gene, from ART-naïve genetic sample. However, the surveillance analysis for acute and/or early infection of HIV-1 could only be restricted to low-density samples. Therefore, to exclude the all potential un-recent or late-chronic infections in our study, we chose the molecular analysis algorithm which might distinguish recent infection from long-standing infection (predictive value 98.7%).^[20,21]^

### 2.3 Phylogenetic subtyping and evolutionary analysis

Blood samples were collected within 3 months after infection had been confirmed. HIV-1 genome RNA was extracted from 200 mL of stored plasma specimens using the QIAmp Viral RNA Mini kit (Qiagen, Valencia, CA) as Manufacturer's instructions. Reverse transcription and nested polymerase chain amplification for partial genes of *pol* were performed by a home brew PCR procedure. A onetube reverse transcriptase polymerase chain reaction kit (Gold-Script one-step RT-PCR kit, Life Technologies), and PCR kit (TaKaRa Ex Taq Kit, Takara Biotechnology Co, Ltd; Dalian, China) were used according to the manufacture's recommendations for amplification of the HIV-1 *pol* gene (protease 1–99 amino acids and part of reverse transcriptase 1–254 amino acids). About 1050-bp *pol* and 660-bp fragments were amplified. The PCR amplification was carried out in a thermal cycler (GeneAmp PCR System 9700, Applied Biosystems). PCR products were directly sequenced in both directions with sequencing primers using ABI 3730 sequencer. Pre-PCR and post-PCR areas are strictly separated to avoid contamination from amplicon aerosol.^[22]^ Multiple alignments were made using Gene Cutter (http://www.hiv.lanl.gov/content/index), with the selected reference sequences of various subtypes/recombinants from the Los Alamos HIV-1 database. A phylogenetic tree was constructed by the neighbor-joining method implemented by MEGA version 6.0 with the Kimura two-parameter and 1000 replicates for bootstrap analysis. New intersubtype or inter-CRF sequences were analyzed by the recombination identification programs (RIP http://www.hiv.lanl.gov, and SimPlot 3.5.1 software). Selection of the clustering sequences was analyzed by Maximum likelihood phylogeny with Fasttree v2.1.7. Local support values were computed with the Shimodaira-Hassegawa test. The node SH-like support value of a cluster >0.9 was considered credible.

To estimate the time of the most recent common ancestor (tMRCA) and the effective population sizes for both CRF01_AE and CRF07_BC strains circulating in Jiangsu, we selected the sequences close to node of each single epidemic cluster in Fasttree ML tree and performed Bayesian inference analysis using the Markov Chain Monte Carlo (MCMC) approach with chain length 200 million in BEAST v1.6.2. An HKY substitution model and uncorrelated relaxed log-normal molecular clock model and Bayesian skyline model were selected for the best-fit model for this analysis. The first 25% of states of each run were discarded as burn in.

The convergence of parameters was checked using Tracer v1.5. The Maximum Clade Credibility (MCC) tree was constructed by TreeAnnotator v1.7.2 and shown by FigTree v1.3.1. The effective sample size (ESS) was >200.

### 2.4 Statistical analysis

The demographic characteristics of these studied subjects were analyzed based on HIV-1 genetic subtypes. Proportions were used for categorical variables. The *χ*^2^ test (or Fisher exact test where necessary) was used to compare proportions between different groups. Data of age-associated and genetic distances were described by the mean ± standard deviation. The trend *χ*^2^ test was used to assess whether there was a change in the trend of the proportions of subtype over time. All tests were 2-tailed and *P* values <0.05 were considered as statistically significant. Statistical analyses were conducted using SPSS20.0 software package (IBM Company, New York).

## 3 Results

### 3.1 Demographic characteristics

**Figure 1:**
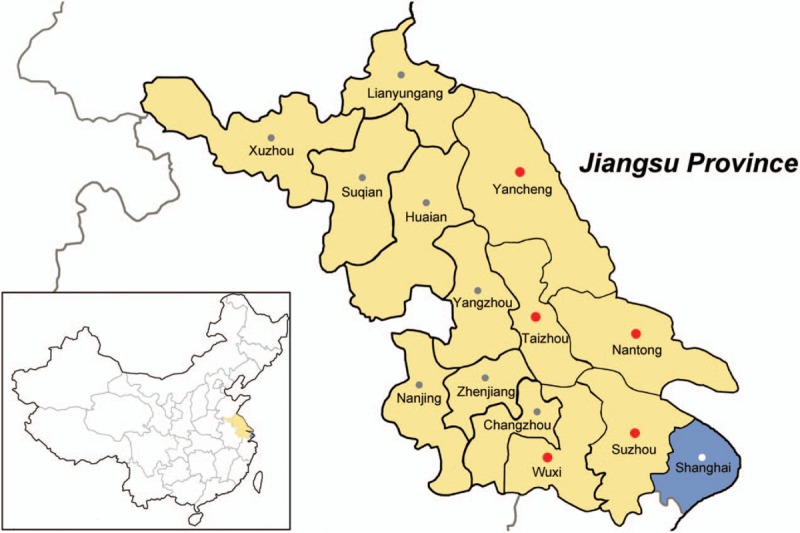
Map of Jiangsu Province, China. A total of 323 HIV-1-infected men who have sex with men individuals were enrolled in 5 major cities of Jiangsu (Suzhou n = 71, Wuxi n = 154, Nantong n = 55, Taizhou n = 21, Yancheng n = 22).

A total of 323 HIV-1-infected MSM individuals were enrolled in 5 major cities of Jiangsu (Suzhou n = 71, Wuxi n = 154, Nantong n = 55, Taizhou n = 21, Yancheng n = 22) including southern, middle, and northern parts of the whole province (Fig. 1). The median age of participants was 27 years, ranging from 18 to 67. Just over 1 in 5 (20.7%) participants had a history of sexually transmitted diseases (STDs). The great majority (99.1%) were Han ethnic and over half (55.1%) were single. Over 2 in 5 (41.5%) had tertiary education. Students accounted for 5.9% (Table 1). Year of diagnosis, ages, and education were considered as significant different stratified by HIV subtype.

**Table 1:**
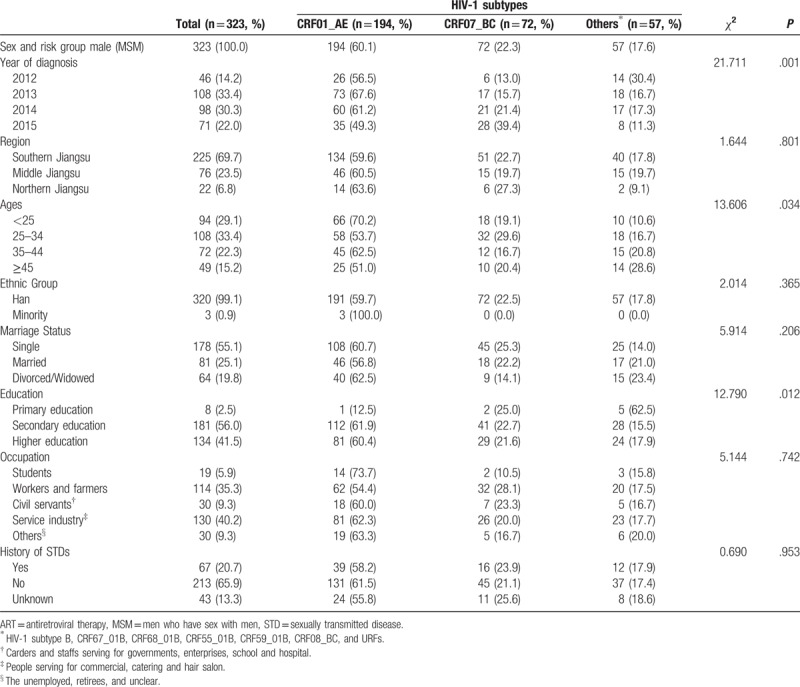
Demographic characteristics of ART-naïve MSM recently infected by HIV-1 in Jiangsu stratified by subtype.

### 3.2 HIV-1 CRF01_AE dominating MSM epidemic

A phylogenetic tree was reconstructed using the Markov Chain Monte Carlo (MCMC) approach with 207 *pol* sequences, consisting of 194 Jiangsu MSM sequences and 13 CRF01_AE reference sequences isolated in Thailand, Australia, and African countries including Rwanda and Uganda. The MCC tree demonstrated all of 194 Jiangsu MSM sequences were segregated into 4 major distinct clusters (indicated as cluster 1–4) with posterior probability value >98% (Fig. 2). Among the 4 clusters, cluster 1 had 172 members that isolated from southern part of Jiangsu (Suzhou & Wuxi, 68.6%, 118/172), middle part of Jiangsu (Nantong & Taizhou, 23.8%, 41/172) and Northern part of Jiangsu (Yancheng, 7.6%, 13/172). For cluster 2, 14 sequences were mainly isolated from Southern and middle parts of Jiangsu (85.7%, 12/14). Cluster 3 and 4 contained 2 and 6 sequences from southern part of Jiangsu, respectively (Table 2).

**Figure 2:**
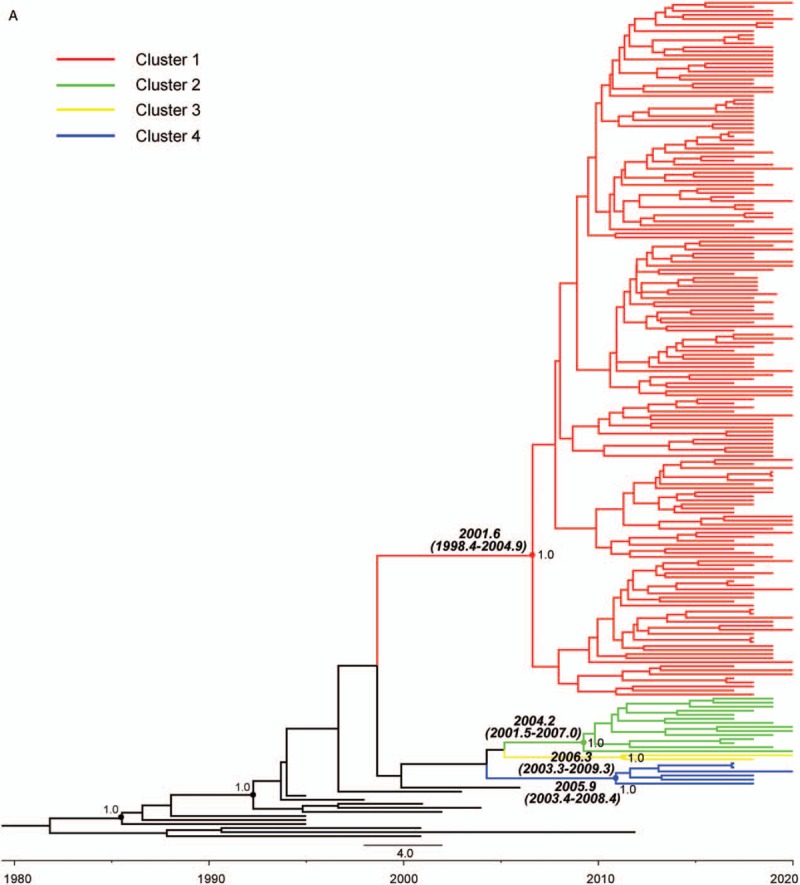
Maximum clade credibility tree representing the rooted genealogy of CRF01_AE and CRF07_BC among recently infected antiretroviral therapy -naïve men who have sex with men in Jiangsu, China, 2012 to 2015. (A) The maximum clade credibility tree for CRF01_AE strain. HIV-1 A1 sequences from Uganda (UG), Rwanda (RW), Australia (AU), and HIV-1 CRF01_AE sequences from Thailand (TH) were used as the references. Cluster 1, 2, 3, and 4 represent 4 clusters belonging to CRF01_AE strain. (B) The maximum clade credibility tree for CRF07_BC strain. HIV-1 subtype C sequence from Indian (IN) and CRF07_BC sequence from Xinjiang, China (CNEF) were used as the references. The branch lengths in the maximum clade credibility trees reflect time and corresponding time-scale is shown at the bottom of the trees. Both the posterior probabilities and the time of the most recent common ancestor for the key nodes are indicated.

**Figure 2 (Continued):**
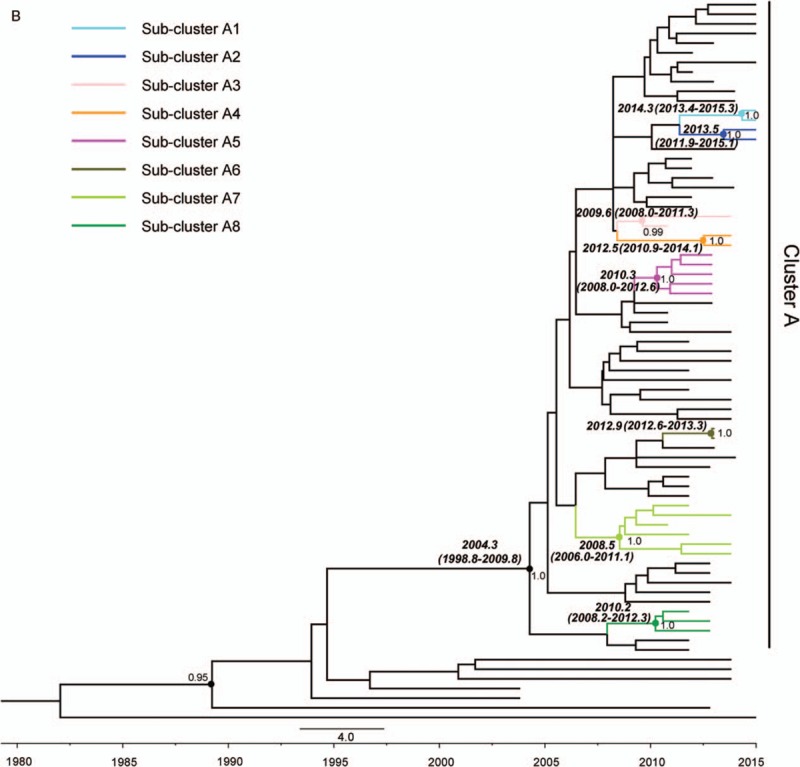
Maximum clade credibility tree representing the rooted genealogy of CRF01_AE and CRF07_BC among recently infected antiretroviral therapy -naïve men who have sex with men in Jiangsu, China, 2012 to 2015. (A) The maximum clade credibility tree for CRF01_AE strain. HIV-1 A1 sequences from Uganda (UG), Rwanda (RW), Australia (AU), and HIV-1 CRF01_AE sequences from Thailand (TH) were used as the references. Cluster 1, 2, 3, and 4 represent 4 clusters belonging to CRF01_AE strain. (B) The maximum clade credibility tree for CRF07_BC strain. HIV-1 subtype C sequence from Indian (IN) and CRF07_BC sequence from Xinjiang, China (CNEF) were used as the references. The branch lengths in the maximum clade credibility trees reflect time and corresponding time-scale is shown at the bottom of the trees. Both the posterior probabilities and the time of the most recent common ancestor for the key nodes are indicated.

**Table 2:**
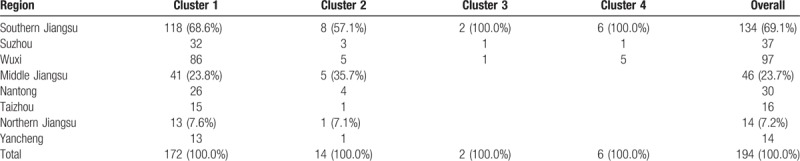
Distribution of CRF01_AE (cluster 1 to 4) in different regions of Jiangsu, China.

**Figure 3:**
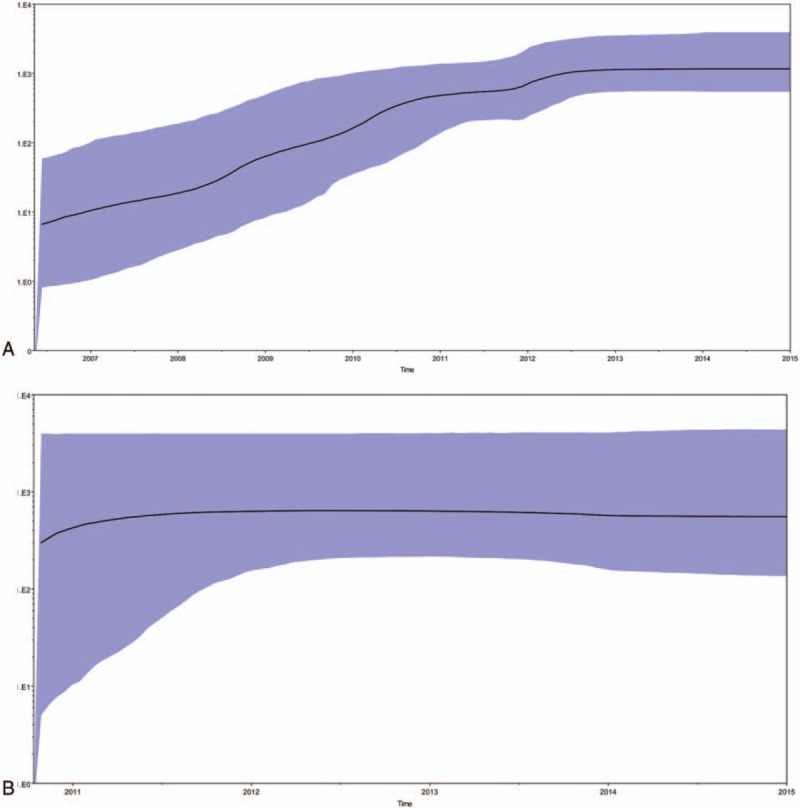
Bayesian skyline plot of HIV-1 CRF01_AE and CRF07_BC *pol* sequences from men who have sex with men in Jiangsu, China, 2012 to 2015. (A) Bayesian skyline plot was estimated to reconstruct the demographic history of CRF01_AE among recently infected antiretroviral therapy-naïve MSM in Jiangsu, China, 2012-2015. 3B: Bayesian skyline plot was estimated to reconstruct the demographic history of CRF07_BC among Recently Infected antiretroviral therapy-naïve men who have sex with men in Jiangsu, China, 2012-2015.

The estimated dates of introduction of cluster 1 into all parts of Jiangsu were 2001.6. Cluster 2 into Jiangsu was dated back to 2004.2. Cluster 3 and 4 had an estimated tMRCA around 2006.3 and 2005.9 in Southern part of Jiangsu, respectively. Bayesian skyline plot (BSP) analysis was also used to infer the estimation of the effective population size at the time of CRF01_AE epidemics among recently infected MSM in Jiangsu, as sampling time span ranged from 2012 to 2015. The demographic history from *pol* BSP identified 4 epidemic growth phases, an initial stable growth phase from the year 2007 to 2011, reached to a platform phase till 2012, followed by an exponential growth phase till 2013 and a stationary phase, approaching the present time. (Fig. 3)

### 3.3 HIV-1 CRF07_BC dominating MSM epidemic

Similarly, the MCC tree was reconstructed using the MCMC approach with 75 *pol* sequences, including 72 Jiangsu MSM sequences and 3 CRF07_BC reference sequences from Los Alamos HIV sequence database. The tree demonstrated 4 (Suzhou n = 3, Nantong n = 1) of 72 Jiangsu sequences (5.6%), which were scattered among each other, whereas the other 68 sequences were segregated into 1 major distinct cluster (indicated as cluster A) with posterior probability value >98% (Fig. 2). Inside cluster A, at least 8 subclusters (indicated as subcluster A1–A8) were found in 24 patients, respectively.

Bayesian inference analysis for the first time indicated the time of introduction for CRF07_BC strain into MSM was around 2004.2 and the estimated tMRCA of 8 subclusters were from 2008.5 to 2014.3. Bayesian skyline plot (BSP) analysis indicated that 2 epidemic growth phases of the effective population size at the time of CRF07_BC epidemics among newly infected MSM in Jiangsu, an initial exponential growth phase till the year 2012, before a relative stationary phase, approaching the present time. (Fig. 3)

## 4 Discussion

To the best of our knowledge, this is the first phylodynamic study depicting the spatiotemporal dynamics, making inference for the effective population size at the time of HIV-1 CRF01_AE/07_BC among newly infected ART-naïve MSM in Jiangsu, China, and to trace the dates of origin for both strains. Jiangsu, a major province of economic prosperity, with well-developed transportation and communication infrastructure and diverse cultures, attracts an increasing number of migrants. By the end of 2015, >79.73 million people are permanent residents in Jiangsu and over 1 in 3 are migrant resident population. In addition, MSM continue to be the population group at higher risk of acquiring HIV-1 infections in developing countries. Well-improved sense of stigma and discrimination for MSM made them flock into metropolises or big cities, where a closely larger social and sexual network more or less had been established or could be easily constructed. Different epidemiological studies worldwide had revealed some risk factors for HIV acquisition in MSM at individual levels, such as unprotected anal intercourse, high frequency of sexual activity with multiple male sex partners, and high prevalence of STDs among this population.^[23,24]^ Therefore, it is worthwhile to note that MSM are currently vulnerable to HIV infection in Jiangsu.

In Western Europe, the incidence of HIV-1 among MSM has increased during the last decade, probably because of an increase in unsafe risk sexual practices and the re-emergence of several sexually transmitted infections (STI) among the young, low-educated, and HIV-unaware individuals.^[25–29]^ The HIV epidemic in European countries such as Italy was mainly attributed to the HIV-1 B subtype.^[30]^ In Europe, national surveillance data showed an increasing proportion of HIV-1 cases among MSM, ranging from 15% in 1996 to 1997 to 22% in 2006 to 2007.^[25]^ However, in Southeast Asia and China, CRF01_AE had quickly overtaken subtype B among MSM during past few years.^[9–11]^ Besides, CRF07_BC strain, the other dominating subtype among the injection drug user (IDU) risk group,^[12–14]^ was also reported to be spreading among MSM. Furthermore, the subtype of HIV-1 among MSM in Jiangsu, mainly were CRF01_AE (63.6%), followed by CRF07_BC (18.2%), B (9.1%), and CRF08_BC (9.1%).^[6]^

Our phylogenetic analysis based on *pol* gene clearly revealed the presence of viral subtype diversity among the studied subjects covering all ages. Obviously, the major HIV-1 subtype is still CRF01_AE, reaching 67.6% in 2013, followed by 15.7% for CRF07_BC, the second dominant strain. In this study, we also determined at least 36 recently identified CRFs and 2 URFs as well, indicating the viral genetic heterogeneity and subtype/recombinant complexity among MSM epidemic in this province. It is well known that CRF01_AE caused an outbreak among the high-risk heterosexual population in Thailand in the late 1980s,^[31,32]^ and was subsequently disseminated to various risk populations in neighboring countries, including China.^[9,33,34]^ Of note, many of CRF01_AE-based inter-subtype or inter-recombinants such as CRF55_01B, CRF59_01B, CRF67_01B, CRF68_01B, and some URFs^[15,18,19,35]^ have been identified recently in different provinces in China, suggesting the future epidemic will even be broader. These new type recombinant strains could be an alert for a future epidemic.

The *pol* region genomic sequencing helps to understand HIV-1 genetic diversity and contributes to the fields of HIV epidemiology, diagnosis, pathogenesis, and vaccine development. Analysis of tMRCA using molecular clock principle can be used to estimate when the viral epidemic began and to estimate the early growth rate.^[36]^ The analyses using phylogenetic reconstruction and Bayesian inference indicate that the spread of CRF01_AE in Jiangsu MSM involved at least 4 viral lineages. Cluster 1 strains play a dominant role in the CRF01_AE epidemic in Jiangsu, and cause a significant proportion of infections in Southern Jiangsu (88.0%), Middle Jiangsu (89.1%), and particularly Northern Jiangsu (92.9%). Cluster 2 was prevalent in Southern Jiangsu (6.0%) and Middle Jiangsu (10.9%). Both cluster 3 and cluster 4 are found mainly in Southern Jiangsu and are responsible for about 1.5% and 4.5% of infections, respectively. (Table 2) We found that tMRCA of CRF01_AE among MSM in Jiangsu was in 2001.6 (cluster 1), 2004.2 (cluster 2), 2005.9 (cluster 4), 2006.3 (cluster 3), a little later than other previous study,^[37]^ in which tMRCA of CRF01_AE among MSM in China was estimated from mid to late 1990s. In addition, we found that the tMRCA of CRF01_AE cluster 1 (2001.6) and 2 (2004.2) was earlier than CRF07_BC (2004.2), indicating an earlier introduction of CRF01_AE strain than CRF07_BC strain into MSM in Jiangsu. Surprisingly, 8 independent subclusters (posterior >0.98) contained within major cluster (A) in MCC tree for CRF07_BC were observed (Fig. 2), implying that a divergent evolution of CRF07_BC strain has occurred, and 8 independent transmission networks might have been established after entering MSM in this province. In our study, for the first time we determined the tMRCA of both CRF01_AE and CRF07_BC strains, and identified multiple epidemic subclusters of CRF07_BC strain circulating among MSM in Jiangsu. However, these epidemic clusters and/or subclusters in relation to their origin of virus introduction, social and sexual network, as well as cross-transmission between different high-risk groups would deserve further investigation in the future study.

Given that the MSM blood samples were from the five local CDCs involving southern, middle, and northern parts of Jiangsu, our results were rather convincing. Nonetheless, a comment should be made about the limitation of the data acquisition in our study. Although the CRF01_AE/07_BC *pol* sequences from all parts of Jiangsu Province were included when this study was initiated, the number of the sequences was relatively small, especially for Northern Jiangsu. Until more archival specimens from the region and more population besides MSM, such as female sex workers and IDU, were retrieved and thoroughly analyzed, the complex dissemination of CRF01_AE/07_BC lineages were interpreted with caution.

In conclusion, based on the laboratory and data analysis skills of molecular epidemiology and the latest available data on MSM from Jiangsu Province, we have found that HIV prevalence remains high and therefore remains a public health priority. Our results demonstrate HIV-1 subtype diversity among ART-naïve MSM recently infected with HIV-1 in Jiangsu, highlighting the need for surveillance of HIV-1 viral diversity and phylodynamics of epidemic cluster to understand the monitoring of the epidemic and control of transmission, improvement of antiretroviral therapy strategies, and design of vaccines.
